# Guanylate binding protein-1 mediates EGFRvIII and promotes glioblastoma growth *in vivo* but not *in vitro*

**DOI:** 10.18632/oncotarget.7109

**Published:** 2016-02-01

**Authors:** Qing Lan, Aidong Wang, Yanwei Cheng, Akitaki Mukasa, Jiawei Ma, Lei Hong, Shuye Yu, Lili Sun, Qiang Huang, Benjamin Purow, Ming Li

**Affiliations:** ^1^ The Experimental Center, the Second Affiliated Hospital of Soochow University, Suzhou, Jiangsu Province, China; ^2^ Department of Neurosurgery, the Second Affiliated Hospital of Soochow University, Suzhou, Jiangsu Province, China; ^3^ Department of Neurology, the Second Affiliated Hospital of Soochow University, Suzhou, Jiangsu Province, China; ^4^ Department of Life Sciences, Luoyang Normal University, Luoyang, Henan Province, China; ^5^ Department of Neurosurgery, the University of Tokyo, Bunkyo-ku, Tokyo, Japan; ^6^ Department of Neurology, University of Virginia, Charlottesville, VA, USA

**Keywords:** GBP1, glioblastoma, EGFRvIII, tumor growth, survival

## Abstract

Glioblastoma multiforme (GBM) is the most common and deadly primary brain tumor in adults. Epidermal growth factor receptor (EGFR) is frequently amplified and mutated in GBM. We previously reported that Guanylate binding protein-1 (GBP1) is a novel transcriptional target gene of EGFR and plays a role in GBM invasion. Here we demonstrate that GBP1 can also be induced by EGFRvIII at the transcriptional level through the p38 MAPK/Yin Yang 1 (YY1) signaling pathway. Silencing of GBP1 by RNA interference significantly inhibits EGFRvIII-mediated GBM cell proliferation *in vitro* and in a mouse model. Overexpression of GBP1 has no obvious effect on glioblastoma cell proliferation *in vitro*. In contrast, in an orthotopic glioma mouse model GBP1 overexpression significantly promotes glioma growth and reduces survival rate of glioma-bearing mice by increasing cell proliferation and decreasing cell apoptosis in tumor. Clinically, GBP1 expression is elevated in human GBM tumors and positively correlates with EGFRvIII status in GBM specimens, and its expression is inversely correlated with the survival rate of GBM patients. Taken together, these results reveal that GBP1 may serve as a potential therapeutic target for GBMs with EGFRvIII mutation.

## INTRODUCTION

Glioblastoma multiforme (GBM) is the most common and most malignant brain tumor, accounting for ∼20% of all the intracranial tumors in adults. GBM is characterized by infiltrating growth, necrosis, robust angiogenesis, and marked genetic heterogeneity [1]. These features make complete surgical removal impossible. As a result, GBM patients' medial survival is typically 12 to 16 months, and the 5-year survival rate is only 3-5% [2].

Like other cancers, multiple genetic and chromosomal alterations are involved in gliomagenesis. The most common genetic lesions associated with GBM are amplification/overexpression and mutation of the epidermal growth factor receptor (EGFR) gene, which are present in 40-60% of GBMs. The most common mutant form of EGFR, EGFR variant vIII (EGFRvIII), is found at 20-30% frequency in GBM. EGFRvIII harbors an in-frame exon 2-7 deletion that leads to constitutive activation of downstream signaling [3,4]. Recently it was determined that EGFR amplification and mutation is more common in the classical subtype of GBM [5]. It has been shown that EGFRvIII plays a key role in GBM tumorigenicity, invasion, chemo- and radio-resistance and often correlates with poor prognosis [6, 7, 8]. Since EGFRvIII is rarely found in normal tissue, it is an ideal therapeutic target against GBM. However, efforts at targeting the EGFRvIII using small molecule inhibitors or antibodies have shown disappointing efficacy in clinical trials for GBM [9].

To better understand the downstream pathways and effector molecules of EGFR signaling, we did oligonucleotide microarray and identified Guanylate binding protein-1 (GBP1) as a novel target gene induced by wtEGFR activation [10]. GBP1 is an interferon inducible large GTPase which has antiviral and antibacterial properties. We found that EGFR promotes GBP1 expression through a unique p38 MAPK/YY1 pathway distinct from the mechanisms of interferon-induced GBP1 expression via Stat1 [11,12]. The induction of GBP1 is essential for EGFR-mediated matrix metallopeptidase-1 (MMP1) expression and glioma cell invasion *in vitro* and *in vivo* [10].

In this report, we demonstrate that GBP1 can also be induced by EGFRvIII activity through the p38 MAPK/YY1 signaling cascade in GBM cells. From a clinical perspective, we found that GBP1 expression is positively correlated with EGFRvIII status in GBM specimens. GBP1 overexpression appears to have no obvious effect on GBM cell proliferation *in vitro*. In contrast, we show for the first time that overexpression of GBP1 enhanced glioma growth in a mouse xenograft model and significantly reduced survival time of glioma-bearing mice. Importantly, silencing of GBP1 using RNA interference markedly inhibited EGFRvIII-mediated GBM tumor growth in mice, suggesting that GBP1 might serve as a potential therapeutic target for treatment of the large percentage of GBMs with the EGFRvIII mutation.

## RESULTS

### EGFRvIII induces GBP1 expression in glioblastoma cells

**Figure 1:**
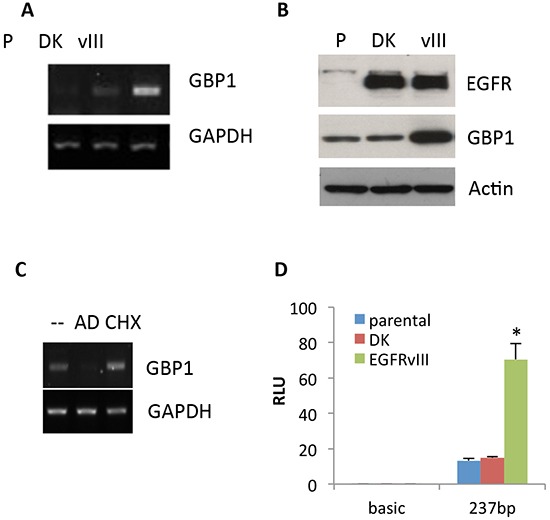
EGFRvIII promotes GBP1 expression The expression of GBP1 was analyzed by semiquantitative RT-PCR **A.** and Western blot **B.** in U87-parental (p), U87-DK and U87-EGFRvIII cells which were serum starved for 48 hr. **C.** After 24 h of serum starvation, U87-P, U87-DK and U87-EGFRvIII cells were treated with AD or CHX for an additional 24 h. *GBP1* and *GAPDH* mRNA was measured by RT-PCR. **D.** U87-P, U87-DK and U87-EGFRvIII cells were transfected with pGL3-237 and pRL-TK for 24 h and then serum starved for 24 h. Firefly and Renilla luciferase activities were measured, and promoter activity is presented as the fold induction of RLU (values of firefly luciferase unit/values of Renilla) as compared with control. The results are expressed as the mean of three independent experiments ± SD. *, *P* < 0.01.

We first examined the impact of EGFRvIII expression on GBP1 expression in glioblastoma cells. To accomplish this, we measured the messenger RNA (mRNA) and protein expression levels of GBP1 in the glioblastoma cell line U87 (termed U87-parental), and U87 cells engineered with EGFRvIII and kinase dead mutant (DK, as a negative control) by retrovirus transduction (termed U87-EGFRvIII and U87-DK, respectively). It was found that GBP1 expression is significantly higher at both the mRNA and protein levels in EGFRvIII expressing cells as compared to the parental and DK cells (Figure 1A and 1B), suggesting EGFR activation is essential for GBP1 induction.

To determine whether EGFRvIII–induced GBP1 expression was directly regulated at the transcriptional level, U87-EGFRvIII cells were treated with 5 μg/ml actinomycin D (AD; a transcription inhibitor) or 100 nM cycloheximide (CHX; a protein synthesis inhibitor) for 24 h prior to RNA collection. Figure 1C shows that AD prevented the upregulation of GBP1 in U87-EGFRvIII cells, whereas no effect was observed in CHX-treated cells, indicating that EGFRvIII enhanced GBP1 expression at the level of transcription and was independent of *de novo* protein synthesis. To further validate this, we cloned the proximal promoter of GBP1 which can be activated by EGF-stimulated wild-type EGFR in GBM cells [10]. As shown in Figure 1D, EGFRvIII significantly stimulated GBP1 promoter activity (5.03 fold; *P* < 0.01), whereas kinase dead EGFRvIII had no effect on GBP1 promoter activation in U87 cells. These data further confirm that EGFRvIII promotes GBP1 expression in glioma cells at the transcriptional level.

### GBP1 is upregulated and positively correlates with EGFRvIII expression status in GBM specimens

**Figure 2:**
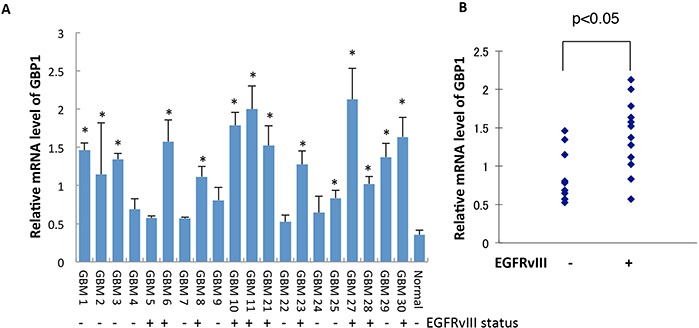
GBP1 is upregulated and correlated with EGFRvIII status in GBM specimens **A.** GBP1 expression in GBM patients was measured by RT-qPCR analysis. Normal denotes normal brain tissue. *, *p* < 0.05. **B.** Correlation analysis between GBP1 and EGFRvIII status in human GBM samples was analyzed for significance using the Kruskal Wallis test; *P* < 0.05 was considered statistically significant.

We then analyzed the mRNA expression profile of GBP1 in a collection of 21 GBM tumor specimens by quantitative RT-PCR (RT-qPCR). The expression status of EGFRvIII in these GBM samples was determined by Western blot using anti-EGFR antibody which can detect both wtEGFR and EGFRvIII (Supplementary Figure S1). Compared with the pooled normal brain tissue, GBP1 expression was significantly elevated in 15 of 25 (75%) GBM samples, and importantly, displayed a positive correlation with EGFRvIII status (*p* < 0.05, Kruskal Wallis test, Figure 2A and 2B), suggesting that EGFRvIII signaling induces GBP1 expression in GBM.

### EGFRvIII promotes GBP1 expression through p38 mitogen-activated protein kinase (MAPK) pathway

**Figure 3:**
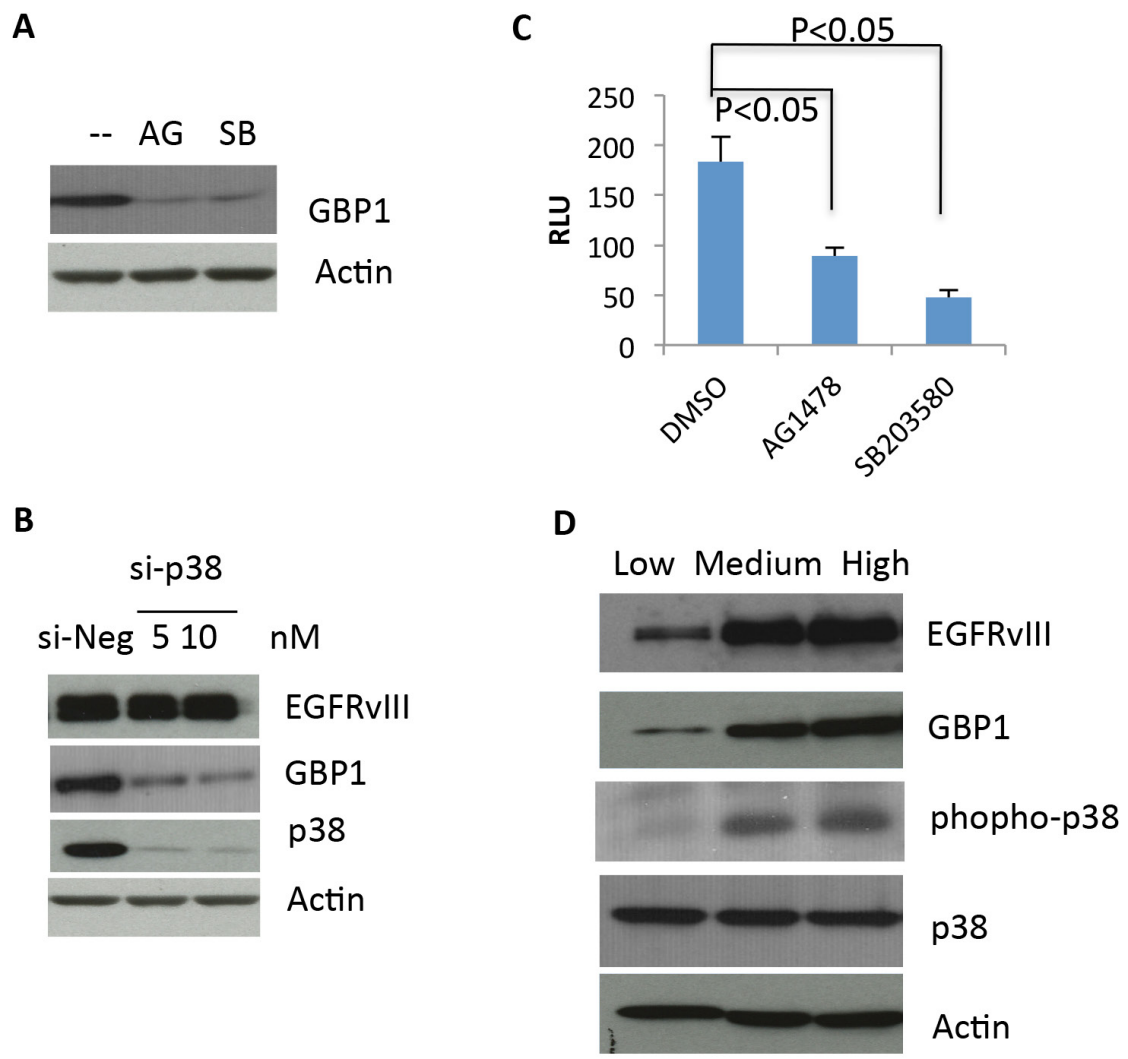
EGFRvIII–stimulated GBP1 expression is p38 MAPK dependent **A.** After 24 h of serum starvation, U87-EGFRvIII cells were treated with DMSO (−), 10 μM of the EGFR tyrosine kinase inhibitor AG1478 (AG), or 20μM of the p38 inhibitor SB203580 (SB) for an additional 24 h before Western blot analysis. **B.** U87-EGFRvIII cells were transfected with the indicated concentration of p38 siRNA (si-p38) or control siRNA (si-Luc) for 24 h and then serum starved for 24 h followed by Western blot analysis. The p38 siRNAs were described previously [10]. **C.** U87-EGFRvIII cells were transfected with pGL3-237 and pRL-TK for 24 h and then serum starved for 24 h. The starved cells were pretreated with DMSO or 20 μM SB203580 for an additional 24 h before reporter assay. This result is expressed as the mean of three independent experiments ± SD. *, *P* < 0.01. **D.** Levels of GBP1, EGFRvIII and phospho-p38 were assessed by Western blot analysis in U87 cells expressing low, medium and high levels of EGFRvIII, which were generated as described elsewhere [25].

We previously reported that EGF-stimulated wtEGFR activation induces GBP1 expression through the p38 signaling cascade [10]. Therefore, we assessed whether EGFRvIII utilizes the same signaling pathway to induce GBP1 expression. To this end, we used both pharmacological (using a specific chemical inhibitor) and genetic (using small interfering RNA [siRNA]) approaches to target EGFR and p38 pathway. We found that inhibition of EGFR activation by AG1478 or of p38 by SB203580 dramatically reduced GBP1 expression in U87-EGFRvIII cells (Figure 3A). Furthermore, knockdown of p38 by siRNA inhibited GBP1 expression in the EGFRvIII expressing cells (Figure 3B). Our promoter luciferase assay also showed that EGFR or p38 inhibition markedly reduced GBP1 promoter activity in the U87-EGFRvIII cells (52% and 75%, respectively, Figure 3C). Consistent with this, a U87 cell model where EGFRvIII is expressed at low, medium and high levels showed a positive correlation between expression of the mutated receptor, phospho-p38, and GBP1 (Figure 3D). Altogether, these data further confirm that EGFRvIII–p38 signaling upregulates GBP1 expression in glioma cells at the transcriptional level.

### YY1 is essential for EGFRvIII-mediated GBP1 expression in GBM cells

**Figure 4:**
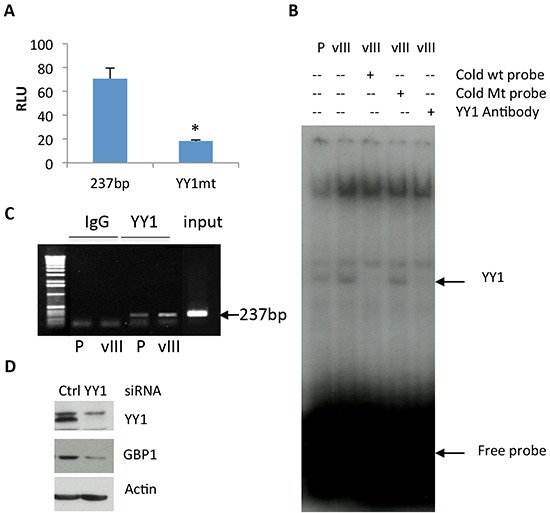
YY1 is involved in regulation of EGFRvIII-mediated GBP1 expression **A.** U87-EGFRvIII cells were transfected with the GBP1 wild-type promoter pGL3-237 or the YY1 deactivated promoter pGL3-237-YY1mut and the internal control pRL-TK for 24 h and then serum starved for 24 h before reporter assay. *, *P* < 0.01. **B.** EMSA analysis. Double-strand YY1 DNA probe was labeled with γ-[32P]ATP and bound to the nuclear extracts of U87-EGFRvIII cells with or without preincubation with a 100-fold excess of YY1 probe or YY1-specific antibody. **C.** ChIP analysis of YY1 element from serum starved U87-EGFRvIII cells using an antibody specific for YY1 or rabbit IgG control. Input chromatin is presented. PCR was performed to amplify the proximal GBP1 promoter (237 bp). **D.** U87-EGFRvIII cells were transfected with YY1 or control siRNA for 24 h and then serum starved for 24 h before Western blot analysis. The siRNAs against YY1 were previously reported in reference [10].

We next sought a downstream transcriptional factor for EGFRvIII-mediated GBP1 induction. We tested if YY1 is involved in this process, since it is required for wtEGFR-stimulated GBP1 expression [10]. The potential YY1 binding motif in the GBP1 promoter was mutated from CCATTTATGG to TTATTTATGG to prevent YY1 binding to the promoter. As shown in Figure 4A, the mutated promoter showed a reduced promoter activation as compared to the intact GBP1 promoter in EGFRvIII-expressing cells (18.3 vs. 70.5).

Electrophoretic mobility shift assays (EMSAs) were performed to test the binding capacity of YY1 motif in the GBP1 promoter. Double-stranded oligonucleotides containing the YY1 motif (−176/−142 bp) were radiolabeled and used as probes of nuclear extracts from U87-EGFRvIII cells as a source of YY1. As would be predicted from the foregoing, EGFRvIII expression in U87 cells increased YY1 DNA binding activity (Figure 4B, first vs. second lane), and the specific band was completely blocked by unlabeled intact but not mutated 100-fold excess of YY1 probe (Figure 4B, third vs. fourth lane). Furthermore, the YY1 antibody disrupted the DNA-protein complex, suggesting that YY1 is indeed at least a component of the DNA binding component detected in the nuclear extracts (Figure 4B, second vs. fifth lane). In agreement with the EMSA results, the chromatin immunoprecipitation (ChIP) PCR assay using a specific antibody against YY1 showed an increased amount of GBP1 promoter DNA with the expected size (237 bp) in U87-EGFRvIII cells as compared to the parental cells (Figure 4C, third vs. fourth lane).

To further verify the role of YY1 in GBP1 induction, we depleted YY1 by siRNA in U87-EGFRvIII cells. Western blot analysis demonstrated that knockdown of YY1 decreased GBP1 protein expression in U87- EGFRvIII cells (Figure 4D). These findings suggested that YY1 functions as a positive regulator of GBP1 expression in the EGFRvIII expressing glioma cells–in surprising contrast to our previous finding that YY1 plays a suppressive role in mediating the effects of wild-type EGFR on GBP1 expression.

### GBP1 is essential for EGFRvIII-mediated GBM cell growth *in vitro* and *in vivo*

**Figure 5:**
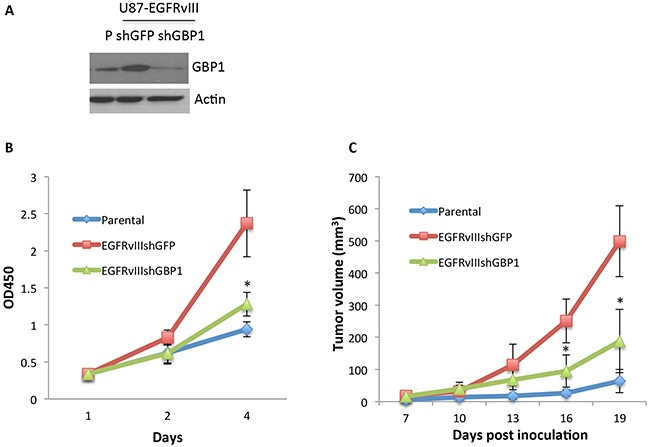
GBP1 is essential for EGFRvIII-expressing GBM cell proliferation in vitro and in vivo **A.** Western blot analysis of GBP1 in U87-Parental and lentiviral shRNA-GFP and shRNA-GBP1 transduced U87-EGFRvIII cells. **B.** WST-1 assay was performed to examine the effect of GBP1 silencing on U87-EGFRvIII cell proliferation. The cells were cultured in DMEM/0.5%FBS condition for the indicated number of days to diminish the effect of serum on cell proliferation. *, *P* < 0.01. **C.** U87-P, U87-EGFRvIIIshGFP and U87-EGFRvIIIshGBP1 cells (5.0×10^5^ cell/mouse, 6 mice/group) were subcutaneously inoculated in nude mice. The tumor volume (mm^3^) was measured at the indicated time in nude mice. *, *P* < 0.05.

Given the important oncogenic role of EGFRvIII in glioblastoma and its relationship with GBP1 expression, we examined the potential role of GBP1 in glioma cell proliferation. A loss-of-function approach was used to assess the role of GBP1 in EGFRvIII-mediated cell growth (Figure 5A). As compared to the U87 parental cells, EGFRvIII expression significantly increased cell proliferation, which was ablated by knockdown of GBP1 (Figure 5B). In contrast, deletion of GBP1 by RNA interference failed to affect U87 parental cell proliferation (Supplementary Figure S2).

To determine if GBP1 is involved in EGFRvIII-mediated GBM tumor growth, U87 parental, U87-EGFRvIIIshGFP and EGFRvIIIshGBP1 were implanted into nude mice. We observed a faster tumor growth rate of the EGFRvIII expressing cells compared to the U87 parental cells. However, silencing of GBP1 expression significantly decreased the tumor growth rate of U87-EGFRvIII cells (Figure 5C), indicating that GBP1 is essential for EGFRvIII-mediated glioblastoma tumor growth.

### GBP1 expression promotes glioblastoma growth in mice

**Figure 6:**
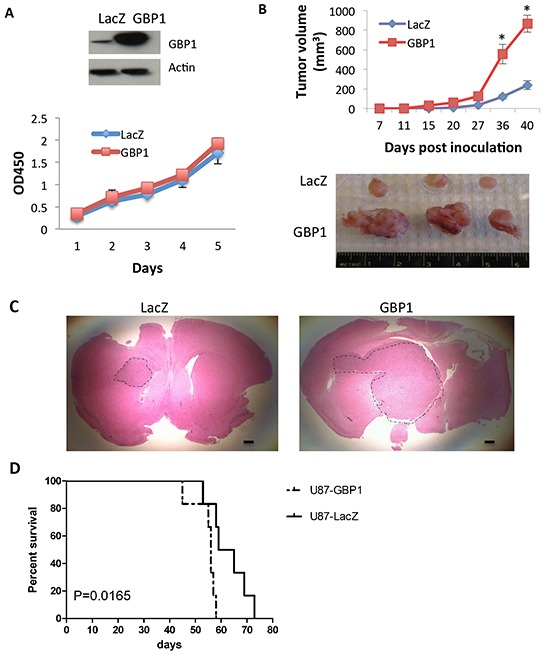
GBP1 overexpression increases glioma tumor growth rate in mice **A.** WST-1 assay was performed to examine the effect of GBP1 overexpression on U87 cell proliferation. Expression level of GBP1 was analyzed by Western blot. **B.** U87-lacZ and -GBP1 flank xenograft tumor volume (mm^3^) was measured at the indicated time in nude mice. 5 × 10^6^ cells/mouse, 6 mice/group. *, *P* < 0.05. **C.** H&E staining of brain sections on day 20 after intracranial inoculation of U87-lacZ (left) or U87-GBP1 (right) (1 × 10^6^ cells/mouse, 6 mice/group). Shown are representative brain slices from tumor-bearing mice. Tumor margins are delineated using a dotted line. Bars, 50 μm. **D.** Kaplan-Meier survival curve of mice after intracranial implantation of U87-LacZ or U87-GBP1 (1 × 10^5^ cells/mouse, 8 mice/group). Statistical comparisons were performed using a log-rank test. *p* = 0.0165.

Finally we asked if GBP1 overexpression alone is able to increase GBM cell growth. We first tested if GBP1 overexpression can increase cell proliferation *in vitro*. GBP1 was overexpressed by retroviral transduction in U87 and A1207 cells, which express low levels of GBP1. We did not observe obvious effects of GBP1 overexpression on the cell proliferation of these two independent cell lines *in vitro* (Figure 6A and Supplementary Figure S3A, S3B). However, when GBP1-overexpressing cells (U87-GBP1) and control cells (U87-LacZ) were subcutaneously implanted into the flank of nude mice, we found that forced expression of GBP1 dramatically increased tumor growth rate compared to the vector control group (Figure 6B). Similar data were obtained from another independent GBP1-overexpressing glioblastoma cell line A1207 (Supplementary Figure S3C). We then stereotactically implanted U87-lacZ and U87-GBP1 cells into the brains of nude mice. Microscopic analysis of brain sections showed that mice implanted with U87-GBP1 cells developed very large tumors, whereas mice implanted with U87-lacZ cells developed smaller tumors (Figure 6C). In addition, Kaplan-Meier survival studies were performed on animals that were intracranially implanted U87-LacZ or U87-GBP1. The average survival time was significantly reduced in the U87-GBP1 group in comparison to the U87-LacZ controls (54.5 vs. 63.8 days, P=0.0165, Figure 6D).

**Figure 7:**
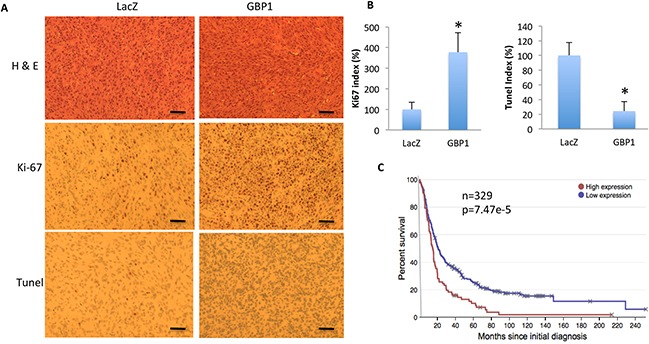
GBP1 overexpression increases cell proliferation and reduces cell apoptosis in mice **A.** Shown are the representative H&E, Ki67 and Tunel staining images of the tumor mass of U87-lacZ (left) and U87-GBP1 (right) on day 20 after intracranial inoculation. Data are representative of two independent experiments. Bars=50 μm. **B.** Quantification of Ki67 and Tunel positive staining cells in the intracranial xenograft tumor. *, *p* < 0.05. **C.** GBP1 expression is inversely correlated with survival rate of GBM patients. Data are adapted from the Rembrandt database (http://rembrandt.nci.nih.gov).

Consistent with this, hematoxylin and eosin (H&E) staining of the xenograft tumor tissues showed increased cancer cell density in the GBP1 expressing tumor. The immunohistochemical (IHC) staining showed that GBP1 expression significantly increased the number of Ki-67 positive cells (3.78 fold versus control) and decreased the number of Tunel-positive cells (20% of control) (Figure 7A, 7B). These results suggested that GBP1 promotes glioma growth *in vivo* partially through increasing tumor cell proliferation and inhibiting cell apoptosis. Consistent with this, the Rembrandt dataset suggests that the expression level of GBP1 is inversely correlated with GBM patients' survival rate (Figure 7C).

## DISCUSSION

In this report, we sought to determine the potential role of GBP1 in EGFRvIII-driven glioblastoma growth. We identified GBP1 as a transcriptional target gene of EGFRvIII signaling. GBP1 expression is positively correlated with EGFRvIII status in GBM patient specimens. Consistent with our results, a recent report using proteomics analysis across a panel of glioblastoma tumor xenografts overexpressing wtEGFR or EGFRvIII identified GBP1 as well as three other proteins (S100A10, major vault protein, and carbonic anhydrase III) to be highly expressed in EGFRvIII expressing xenograft tumors relative to wtEGFR xenograft tumors. Moreover, they found that the increased expression of these four individual proteins is correlated with poor survival in patients with GBM [13]. Functionally, we found that silencing of GBP1 inhibited EGFRvIII-driven glioblastoma cell growth *in vitro* and *in vivo*, suggesting GBP1 is a potential therapeutic target in EGFRvIII-driven tumor growth.

Since wtEGFR and EGFRvIII share the same cytoplasmic domains and downstream signaling cascades, it is not surprising that EGFRvIII utilizes the same signaling cascade p38 MAPK to induce GBP1 expression. Importantly, although YY1 is involved in GBP1 induction in both cases, it acts as an enhancer for EGFRvIII-stimulated GBP1 induction but as a repressor for GBP1 induction by wtEGFR [10]. YY1 can function as a transcriptional activator or repressor depending on the cell type–specific factors, the promoter sequences surrounding the YY1 binding sites, and its cellular environment [14]. It is likely that the persistent signaling from EGFRvIII at a lower intensity [15] is involved in the preferential interaction of YY1 with other transcriptional factors or co-factors, which in turn affects the transcriptional activity of the GBP1 promoter.

GBM is characterized by rapid growth and extensive invasiveness. Our previous report demonstrated that GBP1 is required for EGFR-promoted GBM cell invasion, and GBP1 alone is able to enhance GBM cell invasion through regulating MMP1 expression [10]. The present study demonstrates GBP1 also plays a role in EGFRvIII-expressing cell proliferation *in vitro* and *in vivo*. Interestingly, we did not see obvious effects of GBP1 overexpression on cell proliferation in the cultured cells *in vitro*. However, forced expression of GBP1 promotes glioma growth in mice, and it may act through increasing tumor cell proliferation and reducing cell apoptosis. We propose that GBP1 could enhance glioma growth *in vivo* through indirect mechanisms. It might affect the interaction of GBM cells with the surrounding brain microenvironment, which in turn promotes tumor growth *in vivo*. Further work remains to identify the molecules or cytokines/chemokines involved in these complex cell-cell interactions.

The role of GBP1 in cancer is still controversial. One report showed that GBP1 is downregulated and acts as a tumor suppressor in colorectal cancer cells [16]. In contrast, GBP1 is upregulated and a poor prognostic marker in oral cavity squamous cell carcinoma (OSCC) [17]. Furthermore, GBP1 was shown to be upregulated in paclitaxel-resistant ovarian cancer cells and to confer moderate paclitaxel resistance [18,19]. One recent study reported that GBP1 is a key molecule contributing to radioresistance in multiple cancer cells [20]. The current study and our previous study have provided strong evidence that GBP1 is significantly upregulated in GBM and plays a key role in GBM growth and invasion [10], but the functions of GBP1 in cancer appear to be cell-type specific.

This study demonstrates that increased expression of GBP1 in EGFRvIII-expressing GBMs enhances glioblastoma growth and progression. Since GBP1 belongs to a large GTPase family consisting of 7 members (GBP1-7) in humans [21], it will be interesting to determine if its GTPase activity and other family members are relevant in GBM. It may soon be possible to develop optimal therapeutic strategies (i.e., small molecule inhibitors) targeting GBPs for preclinical and clinical testing in GBM.

## MATERIALS AND METHODS

### Chemicals, plasmids, cell lines and clinical samples

The p38 inhibitor SB203580 was obtained from EMD. N-terminal Flag-tagged GBP1 was cloned as described previously [22] and inserted into retrovirus vector pBABE-puro. pLKO.1-shRNA-GFP and -GBP1 constructs were obtained through the RNAi Consortium shRNA Library (Broad Institute of Massachusetts Institute of Technology and Harvard). The human GBP1 promoter 237 bp (−218/19 bp) was cloned by PCR using specific primers sense (5′-AGCTTCTGGTTGAGAAATCTTTAAACC-3′) and antisense (5′-TGGCTTCTAGCACTTCTGTGTCTCTC-3′) and inserted into the firefly luciferase vector, pGL3 basic (Promega). To generate a construct with mutation of the YY1-binding elements, the QuikChange site-directed mutagenesis kit (Agilent Technologies) was used to change the potential YY1 binding motif from CCATTT to TTATTT. All GBM cell lines were cultured in DME with 10% FBS. GBM samples, provided by R. Nishikawa (Saitama Medical University, Hidaka-shi, Saitama, Japan), were obtained at the time of surgery after written informed consent. Gene Pool human normal brain tissue cDNA was purchased from ThermoFisher Scientific.

### Virus production and infection

293T cells were transfected with pBABE-puro–LacZ, or -GBP1 with pCL10A1 using Lipofectamine 2000 (Invitrogen) to produce retrovirus. 293FT cells were cotransfected by pLKO.1-shRNA-GFP (5′-CAAGCTGACCCTGAAGTTCAT-3′) or -GBP1 (5′-CGGAAATTCTTCCCAAAGAAA-3′) with pCMVDR8.91 and pMD.G-VSV-G using Lipofectamine 2000 to produce lentivirus. Viral supernatants were harvested and filtered (0.45 μm) at 48 and 72 h posttransfection. Glioma cells were infected overnight in the presence of 8 μg/ml polybrene and then selected for 5 d in growth medium containing 1 μg/ml puromycin. The stable clones were verified by Western blot.

### Western blots and antibodies

Cells were lysed in RIPA buffer (150 mM NaCl/1.0% Triton X-100/0.5% Na deoxycholate/0.1% SDS/50 mM Tris, pH 8.0/complete protease inhibitor; Roche). Primary antibodies used were anti-YY1 (c20), anti-p38 (Santa Cruz Biotechnology, Inc.), anti-EGFR (c13; BD), anti–β-actin and –phospho-p38 (Cell Signaling Technology), and anti-GBP1 (MBL International).

### Cell proliferation assay

The WST-1 assay (Roche Diagnostics) was performed to assess the cell proliferation or viability. Briefly, the cells were plated onto a 96-well plate at a concentration of 1500 cells/well and cultured for the indicated days. The WST-1 reagent was added to wells at 1:10 ratio and incubated at 37°C for 2 hr. Absorbance was measured at 450 nm using GENios Pro (Tecan).

### RT-PCR and real-time qPCR

Total RNA was harvested by TRIZOL reagent (Invitrogen) and reverse transcribed (SuperScript II First Strand kit; Invitrogen). Semiquantitative RT-PCR and qPCR were performed as described in our previous report [10].

### EMSA

Double-stranded DNA probes (−175/−142 bp of GBP1 promoter) were labeled with 5 μCi γ-[^32^P]ATP (PerkinElmer) using T4 polynucleotide kinase (Promega). The labeled oligonucleotides were purified from the free γ-[^32^P]ATP using a quick-spin column (Roche) according to the manufacturer's instructions. EMSA procedure was described previously [10].

### Reporter assays

U87 parental cells and U87-EGFRvIII cells were cotransfected with pGL3-237 or pGL3-237yy1mt and with the Renilla luciferase plasmid pRL-TK (Promega). 24 h after transfection, the cells were starved in serum-free medium for 48 h. The Dual-Luciferase Reporter Assay (Promega) was performed according to the manufacturer's instructions, and values were read on a GENios Pro (Tecan).

### ChIP-PCR

The assay was performed as previously described [23, 10]. Briefly, U87 parental and U87-EGFRvIII cells were treated with 1% formaldehyde to cross-link proteins to DNA. The cell pellets were resuspended in lysis buffer and sonicated to yield a mean DNA size of 500 bp. Sonicated extracts were precipitated with 1 μg of normal rabbit IgG or rabbit anti-YY1 antibody overnight at 4°C. The cross-linked DNA–protein complexes were reversed by heating at 65°C. The purified DNA was then subjected to PCR to amplify the GBP1 promoter regions using the same specific primers for GBP1 promoter cloning.

### Xenograft model and histological analysis

All animal experiments conformed to ethical principles and guidelines approved by the Second Affiliated Hospital of Soochow University Institutional Animal Care and Use Committee. For the subcutaneous model, 5×10^6^ U87-lacZ or-GBP1 cells, or 5×10^5^ U87, U87-EGFRvIII-shGFP or U87-EGFRvIII-shGBP1 in 100 μl PBS were implanted into the flank of the 4–5-wk-old athymic nude mice (6 mice/group). Tumor size was measured at the indicated time intervals with a caliper, and tumor volume (V) was calculated using the formula V = [1/2] ab^2^, where a and b are the long and short diameters of the tumor, respectively. For the intracranial model, 1×10^6^ of U87-lacZ or -GBP1 cells in 5μl PBS were injected intracranially into nude mice (6 mice/group) using a guide screw system as described by Lal et al. [24]. After 14–20 d, mice were euthanized, and their brains were removed and embedded in paraffin. For the survival study, 1×10^5^ of U87-lacZ or -GBP1 cells in 5 μl PBS were injected intracranially into nude mice (8 mice/group). The survival and health of the Tumor-bearing mice were closely monitored. Survival data were plotted on a Kaplan–Maier curve. The tumor tissues were sectioned and stained with CD31 (Abcam), Ki67 (Abcam), Tunel (Abcam), and hematoxylin and eosin (H&E) staining.

### Statistical analysis

Correlation analysis between GBP1 and EGFRvIII expression in human glioma samples was analyzed for significance using the Kruskal Wallis test, where *P* < 0.05 was considered statistically significant. For other experiments, results are expressed as the mean ± SD. Statistical analyses were performed by Student's *t* test. *P* < 0.05 was considered statistically significant.

## SUPPLEMENTARY FIGURES


