# Supplementary material for: Guanylate binding protein-1 mediates EGFRvIII and promotes glioblastoma growth in vivo but not in vitro
Source: Oncotarget. 2016 Feb 1;7(9):9680–91. doi: 10.18632/oncotarget.7109 (PMC4891076; doi:10.18632/oncotarget.7109)
Supplement: Supplementary file 1 [file oncotarget-07-09680-s001.pdf]

SUPPLEMENTARY FIGURES

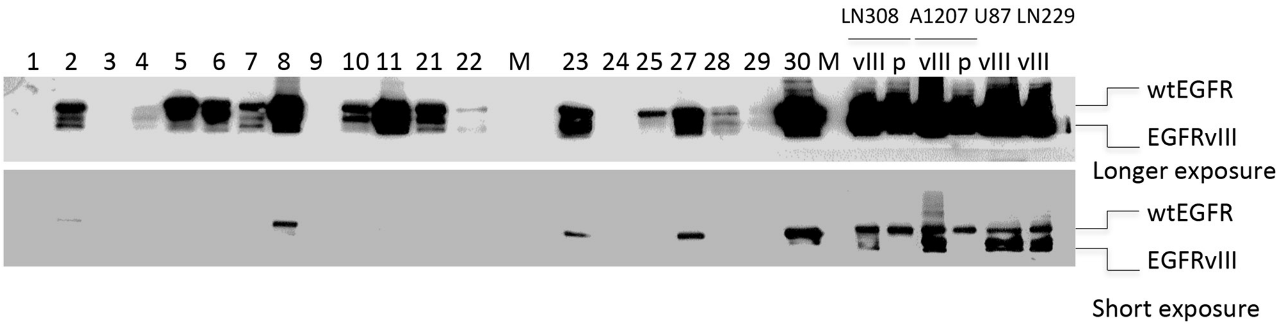

Supplementary Figure S1: Expression status of EGFRvIII in a collection of human GBM clinical samples. Western blot analysis of EGFRvIII was performed in 20 human GBM specimens. LN308-parental (P), LN308-EGFRvIII(vIII), A1207-P, A1207-vIII, U87-vIII, LN229-vIII are shown as positive controls for EGFRvIII expression. M=the protein marker lane.

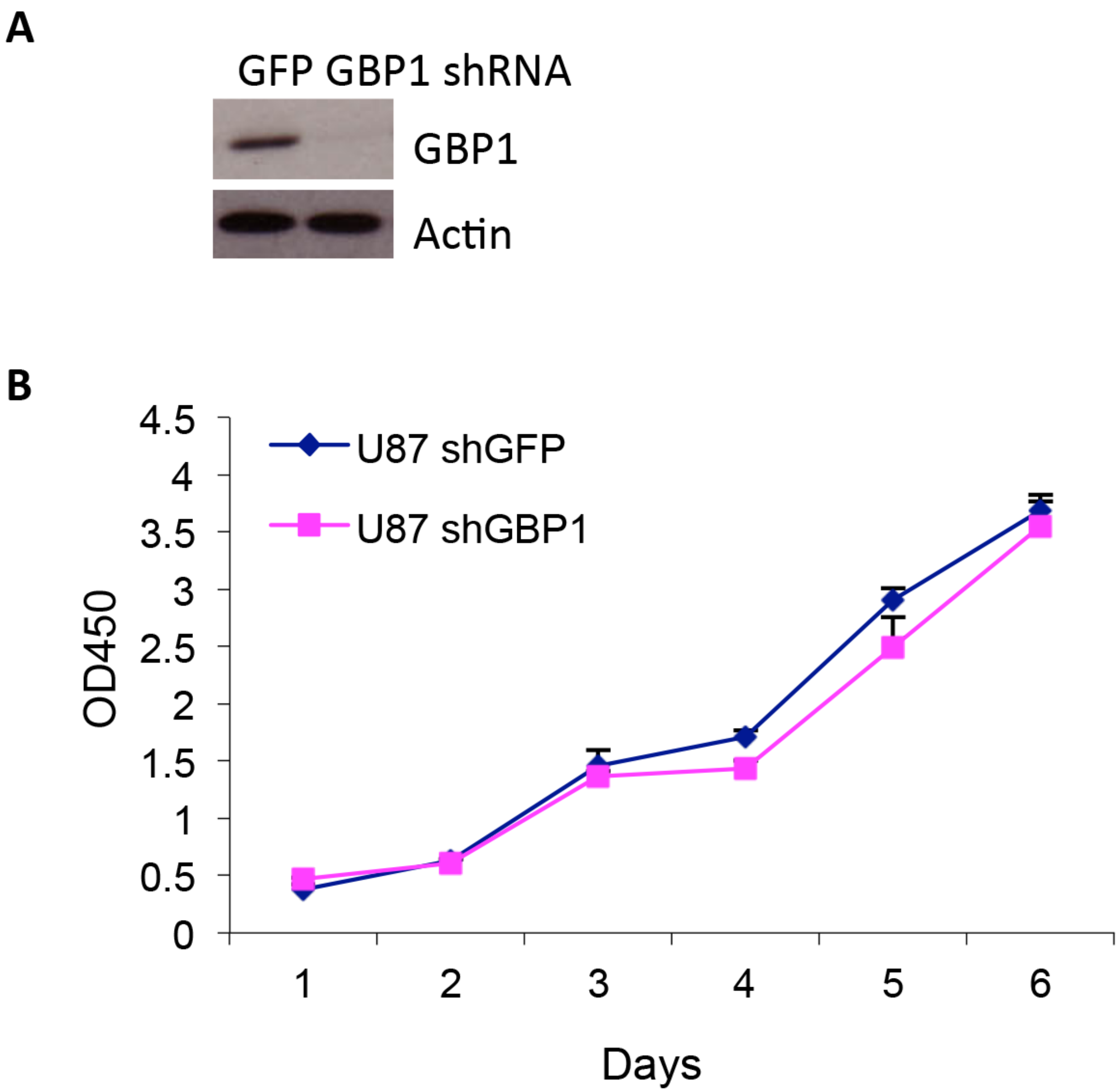

Supplementary Figure S2: Loss of GBP1 expression has no effect on U87 parental cell proliferation. A. Western blot analysis of GBP1 in lentiviral shRNA-GFP and –GBP1 transduced U87 parental cells. **B.** WST-1 assay was performed to examine the effect of GBP1 silencing on U87 parental cell proliferation.

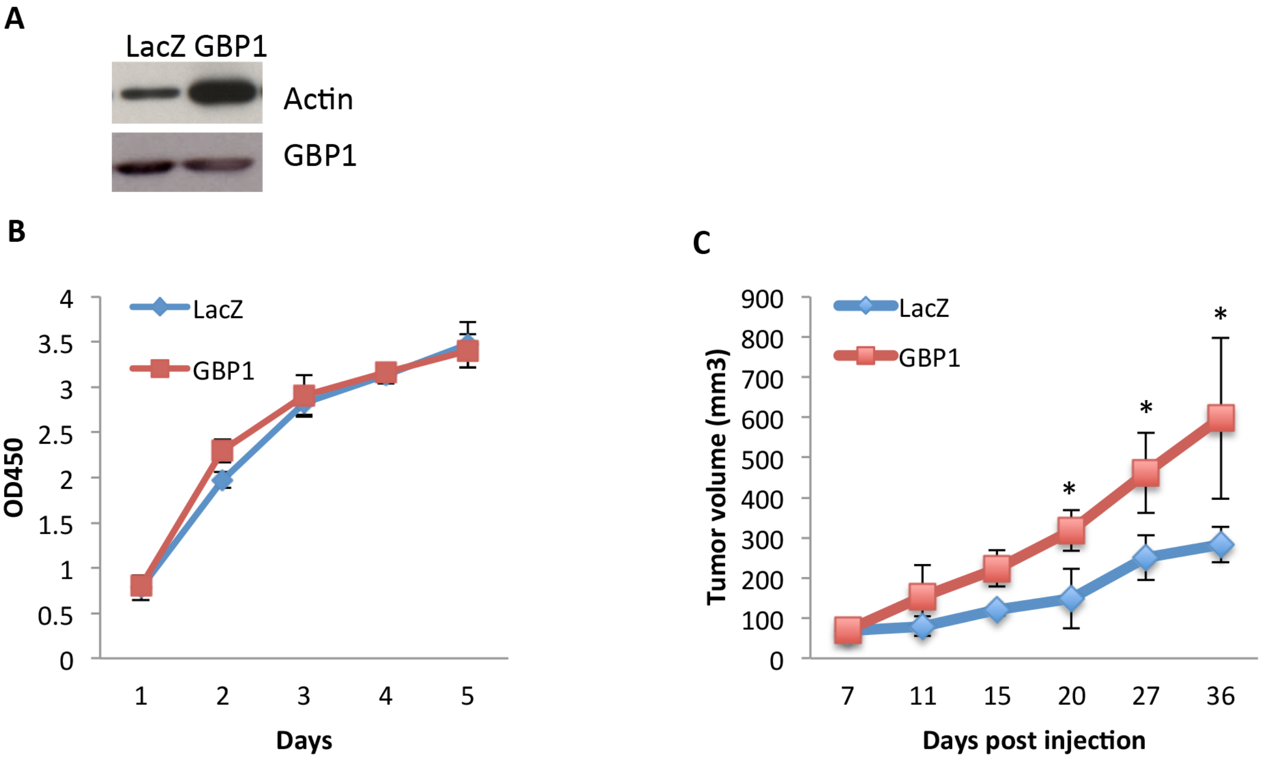

Supplementary Figure S3: Forced expression of GBP1 increases A1207 cell growth *in vivo* but not *in vitro*. A. Western blot analysis of GBP1 in retroviral LacZ- and –GBP1 transduced A1207 cells. **B.** WST-1 assay was performed to examine the effect of GBP1 expression on A1207 cell proliferation in vitro. **C.** A1207-lacZ and A1207-GBP1 flank xenograft tumor volume (mm^3) was measured at the indicated time in nude mice. 5 × 10^6 cells/mouse. 6 mice/group. *, $P < 0.05$.
